# Nosocomial outbreak in a respiratory ward caused by the SARS-CoV-2 Omicron BA 5.2.1 subvariant associated with non-severe illness in vaccinated patients

**DOI:** 10.1017/S0950268823001590

**Published:** 2023-09-26

**Authors:** Guduru Gopal Rao, Shamiso Jinjika, Dianne James, Nyarayi Mukombe, Bharat Patel, Amelie Chietcheu, Christine Macmanus, David Adeboyeku, Emma Davies, Benjamin Brown

**Affiliations:** 1 Departments of Microbiology, Northwick Park Hospital, London, UK; 2 Infection Prevention and Control, Northwick Park Hospital, London, UK; 3 Respiratory Medicine, Northwick Park Hospital, London, UK; 4 Department of Virology, Manchester University NHS Foundation Trust, Manchester, UK; 5 United Kingdom Health Security Agency, UK; 6 Faculty of Medicine, Imperial College, London, UK

**Keywords:** outbreaks, COVID-19, vaccines

## Abstract

In this short report, we describe an outbreak of COVID-19 caused by Omicron subvariant BA.5.2.1 in highly vaccinated patients in a respiratory ward in a large acute general hospital in North West London, United Kingdom. The attack rate was high (14/33 (42%)) but the clinical impact was relatively non-severe including in patients who were at high risk of severe COVID-19. Twelve of fourteen patients had COVID-19 vaccinations. There was only one death due to COVID-19 pneumonitis. The findings of this outbreak investigation suggest that while the transmissibility of Omicron BA.5.2.1 subvariant is high, infections caused by this strain are non-severe in vaccinated patients, even if they are at high risk of severe COVID-19 infection.

Coronavirus disease 2019 (COVID-19) caused by severe acute respiratory syndrome coronavirus 2 (SARS-CoV-2) has evolved into global pandemic and continues to affect many parts of the world [1]. The original strain of SARS-CoV-2 has also continuously evolved as a result of mutations. While many of these mutations are of little consequence, some of these mutants have been termed variants of concern (VOC). The VOC have important public health significance because they have been demonstrated to be associated with one or more of the following changes at a degree of global public health significance. These include an increase in transmissibility or increase in virulence or change in clinical disease presentation or a decrease in the effectiveness of public health and social measures or available diagnostics, vaccines, and therapeutics. The Office of National Statistics (ONS), England, reported that in the week ending 24 June 2022, there was an increase in the percentage of people tested positive for coronavirus (COVID-19) in the United Kingdom and Europe, likely caused by infections compatible with Omicron subvariants BA.4 and BA.5. In England, nearly 1 in 40 people (2.5%) was infected. According to ONS, the infection levels were higher than at the peak of the alpha variant (B.1.1.7) wave in January 2021 [2]. There are surprisingly few published reports of hospital-acquired infection or hospital outbreaks with the Omicron BA.5 subvariant.

At the time of the outbreak (June–July 2022), the most prevalent VOC in the United Kingdom, Europe, and United States were the BA.4 and BA.5 Omicron subvariants of SARS-CoV-2 [3]. BA.5 subvariant accounted for more than 75% of all cases of COVID-19 in the United Kingdom [4]. The incidence of hospital admission rates was lower than those observed during COVID-19 in March–June 2020, which likely reflects the protective effect of previous infection-and/or vaccine-derived population immunity [5]. During the first 6 months of pandemic in England, it was estimated that up to 1 in 6 SARS-CoV-2 infections among hospitalised patients with COVID-19 could be attributed to nosocomial transmission [6]. A study in Wales reported that the inpatient mortality rate for healthcare-associated COVID-19 (HA COVID-19) ranged from 38% to 42% and was consistently higher than that for inpatients with community-acquired COVID-19 (31%–35%). That study also found that patients with HA COVID-19 were old and frail and had more comorbidities than those with community-acquired infections [7].

However, there are only a few reports of HA COVID-19 or outbreaks in hospitals with SARS-CoV-2 variants that have subsequently emerged, especially in the context of vaccinated patients and healthcare workers (HCWs) [8].

In this report, we describe an outbreak of COVID-19 infection caused by the Omicron BA.5 subvariant in a respiratory ward in a large acute general hospital in North West London, United Kingdom, and its impact on patients, nearly all of whom were vaccinated. The outbreak occurred in a 33-bed ward mainly caring for patients with respiratory illnesses and a few patients with non-respiratory illnesses between 27 June and 7 July 2022. HA COVID-19 was first detected in a patient on 27 June when she was routinely screened for COVID-19, 7 days post-admission (PA). The following day, five patients were found to be positive for COVID-19. The tests were done either because the patient was symptomatic or as part of PA screening. An outbreak was declared as two or more cases in a single setting had become symptomatic or were detected on screening on or after day eight of hospital admission. Further patients were detected subsequently with PA hospital stays ranging from 7 to 121 days prior to the detection of COVID-19. All patients on the ward were routinely screened for SARS-CoV-2 using reverse transcriptase polymerase chain reaction (RT-PCR) using GeneXpert (Cepheid, USA) or SAMBA II (DRW, UK) at the time of admission, day 3 PA, day 7 PA, and day 14 PA. This was done as part of London-wide mandated surveillance programme to detect healthcare-associated acquired COVID-19 (HA COVID-19). According to the guidance, COVID-19 infections detected <3 days PA were considered community acquired; COVID-19 detected 3–7 days PA were considered possible HA-COVID-19; COVID-19 detected 8–14 days PA were considered probable HA-COVID-19; and COVID-19 detected after 14 days PA were considered definite HA-COVID-19. The outbreak was investigated and managed by the hospital’s ‘Outbreak Control Team’ consisting of the Infection Prevention and Control Team, microbiologist, senior ward nurses, and medical consultants overseeing the care of patients. Demographic and clinical information regarding patients was obtained from electronic patient record (EPRO). Real-time reverse transcriptase PCR (RT PCR) tests for COVID-19 in patients were performed in an accredited local laboratory at Northwick Park Hospital, London. COVID-19-positive specimens were sequenced at the United Kingdom Health Security Agency (UKHSA).

At the time of the outbreak, the ward accommodation consisted of five bays with en suite facilities (four bays with six patients and one bay with four patients). All the bays had natural ventilation through windows which are kept open for much of the day. Typically, there was one nurse attending to six or seven patients. In addition, there were 5–6 healthcare assistants who worked alongside the nursing staff. The number of doctors visiting the ward was variable before the outbreak. Alcohol-based hand sanitisers were provided at the end of every bed and hand-washing sinks were available in each bay. Regular audits showed good hand hygiene compliance with hospital infection control guidelines, which in turn are based on WHO guidelines. In keeping with the hospital’s infection control guidelines, all COVID-19-positive patients were placed in single rooms or placed in a cohort in one or more bays. Infection control precautions included appropriate personal protective equipment (gloves, apron, and FFP3 mask) by HCWs when caring for positive patients in accordance with national guidance. Enhanced PPE (single-use gown, FFP3 mask, or hood and eye protection or visor) was worn when performing aerosol-generating procedures on positive patients. In our hospital, there were six outbreaks of COVID-19 infection in various wards in the preceding 3 months infecting a total of 47 patients with two associated fatalities.

Following the declaration of the outbreak, all uninfected patients on the ward were screened. Contacts were defined as those who shared the same ward space (six- or four-bedded bays). Vulnerable patients (those at high risk of developing severe COVID-19) who were contacts were screened every 48 h and vulnerable patients who were not contacts were screened on day 3, day 7, and then weekly thereafter. As there was no mechanical ventilation, the ward ensured that natural ventilation in the ward was improved by opening the windows. The importance of hand hygiene was re-emphasised, and the Infection Prevention and Control Team monitored the compliance with infection prevention and control measures on a daily basis. During the outbreak, the frequency of cleaning the ward environment was increased from twice to thrice a day. The ward was closed for new admissions of patients. All positive patients were isolated in side rooms or cohort bays for 10 days (non-immunocompromised patients) or 14 days (immunocompromised patients) or not unless they were discharged to their own homes where there were no vulnerable persons. Patients were not screened prior to discharge. During the closure of the ward, visitors were generally not allowed into the ward. Affected patients and/or their families were informed about the outbreak and the need for control measures to prevent further spread. The ward was reopened for admissions 7 days after the last case on 14^th^ July 2022. All HCWs who were COVID-19 positive were excluded from the hospital for 10 days from the onset of symptoms. Alternatively, HCWs could return to work if the antigen tests were negative on two successive days, starting with 6^th^ day after the onset of symptoms.

Characteristics of affected patients including SARS-CoV-2 lineage, age group, sex, admission diagnosis, location, date of admission, length of stay prior to admission, symptoms, ‘high risk of severe COVID-19’ status according to the UK guidance, COVID-19-specific antiviral treatment, vaccination status, and the outcome of the infection are shown in Table 1. The attack rate of the infection for patients was 14/33 (42%). Specimens from eight patients who were positive for SARS-Cov-2 by PCR were sent to the UKHSA Genomic Sequencing Service. Specimens from the remaining six (patients 1,6,8,10,12,14) were not available for submission to the reference laboratory. Of the 8 samples sent for SARS-CoV-2 sequencing, 2 samples (patients 3 and 9) failed to produce a lineage due to insufficient genome coverage and the remaining 6 samples (patients 2, 4, 5, 7, 11, and 13) were all identified as SARS-CoV-2 lineage BA5.2.1. Patient samples were aligned against 54 Omicron lineage SARS-CoV-2 genomes using MAFFT command line version 7.475 with the subsequent phylogenetic tree generated using IQ-TREE (multicore version 2.0.3) command line with default parameters including ModelFinder and 1,000 ultrafast bootstrap replicates to determine branch confidence. A midpoint-rooted maximum likelihood phylogenetic tree showed that the sample from patient 4 did not cluster with any other patient samples. Samples from patients 2, 5 and 7 are clustered together, with samples from patients 11 and 13 very close on the next node. Both patient 4 and the large patient cluster showed a >96% bootstrap support at their primary branch. Internal branch bootstrap support for the main sample cluster ranged from 73 to 98%; however, no other samples were contained within the cluster. The results of the phylogenetic analysis indicate that transmission from patient 4 to subsequent patients can be excluded. For samples from patients 2, 5, 7, 11, and 13, the similarity of genomes and close clustering on the phylogenetic tree suggests possible transmission occurrence, although the lack of sequences from patients in the same geographical area within the tree means that this cannot be definitely proven. Extra analysis involving more samples from patients not associated with this cluster would help strengthen any association, but this was not possible at this time.

**Table 1.** Characteristics of patients infected with COVID-19

Patient	Age group & Gender	Admission diagnosis/location (Bay, bed number)	High Risk of severe COVID-19^a^	Date positive Hospital stay (days) prior to COVID-19	COVID-19-related symptoms	Treatment for COVID-19	Primary vaccine Number of doses; vaccine manufacturer	Booster Number of doses; vaccine manufacturer	Days since last vaccination at the time of infection	Outcome on 31/07/2022
1	70–80 M	Bronchopneumonia/Side room 1	Yes	27/6/2022 7	Nil	Nil	2x Astra Zeneca	1 x Pfizer	237	Discharged alive
2	70–80 M	Guillain-Barre Syndrome/side room 1	No	29/06/2022 121	Nil	Nil	2x Pfizer	1x Pfizer	223	Discharged alive
3	70–80 F	IECOPD; AKI; thrombocytopenia/D1	Yes	28/06/2022 39	Nil	Remdesivir	2x Astra Zeneca	1 x Pfizer	157	Died 19/7^b^
4	70–80 M	IECOPD/A4	No	28/06/2022 19	Increased shortness of breath and O2 requirement	Remdesivir+ corticosteroids	2x Pfizer	2x Pfizer	94	Discharged alive
5	70–80 M	Hypercalcaemia, PE; Ca. Lung/A6	Yes	28/06/2022 11	Increased shortness of breath and O2 requirement	Remdesivir+ corticosteroids	2x Pfizer	1x Pfizer	248	Discharged alive
6	50–60 M	IE bronchiectasis; pneumonia/A5	Yes	28/06/2022 8	Nil	Remdesivir	2x Pfizer	1x Pfizer +1x Spikevax	46	Discharged alive
7	50–60 M	Rehabilitation after road traffic accident/side room 2	No	28/06/2022 60	Fever	Nil	Not vaccinated	NA	NA	In hospital
8	50–60 F	Asthma; Fever/D2	No	29/06/2022 7	Asthma triggered by COVID-19	Corticosteroids	2x Pfizer	1x Pfizer	213	Discharged alive
9	80–90 M	Empyema/side room 4	Yes	02/07/2023 59	Nil	Nil	2x Pfizer	1x Pfizer +1x Spikevax	85	Discharged alive
10	20–30 M	Low level of consciousness/C3	No	03/07/2023 17	Nil	Nil	2x Pfizer		193	Discharged alive
11	80–90 M	Empyema; Ca. Lung/C2	Yes	03/07/2023 11	Nil	Nil	2x Pfizer	2x Pfizer	92	Discharged alive
12	60–70 M	Fall; Stroke/A1;	No	03/07/2023 16	Nil	Nil	Not vaccinated	NA	NA	Discharged alive
13	80–90 M	LRTI; myeloma, immunosuppression/B2	Yes	05/07/2023 12	Pancytopenia	Remdesivir	2x Pfizer	2x Pfizer	114	Discharged live
14	60–70 M	HAP; hypoxic brain injury	Yes	07/07/2023 20	Pneumonitis but no increased O2 requirement	Nil	2x Pfizer	1x Pfizer +1x Spikevax	58	In hospital

a NHS definition of high risk of severe COVID-19′ status: Patients having any of the following conditions: Down’s syndrome, certain types of cancer (such as a blood cancer like leukaemia or lymphoma), sickle cell disease; certain conditions affecting blood, chronic kidney disease (CKD) stage 4 or 5, severe liver disease, organ or bone marrow transplant, certain autoimmune or inflammatory conditions (such as rheumatoid arthritis or inflammatory bowel disease), HIV or AIDS and have a weakened immune system, a condition affecting the immune system, a rare condition affecting the brain or nerves (multiple sclerosis, motor neurone disease, Huntington’s disease or myasthenia gravis), a severe problem with the brain or nerves, such as cerebral palsy, severe or multiple learning disabilities, a weakened immune system due to medical treatment (such as steroid medicine, biological therapy, chemotherapy, or radiotherapy).

b Death due to COVID-19 pneumonitis.

HA COVID-19 was first detected in a patient on 27 June 2022. The last case among patients and HCWs occurred on 7^th^ July 2022 and 4^th^ July 2022, respectively. No patients were infected for a further 4 weeks after the last infected case, at which time the outbreak was declared to have ended. No analytical epidemiological investigations were undertaken.

Nine HCWs who worked in the ward during the outbreak were also infected. Two HCWs were infected 3–8 days prior to the outbreak. All HCWs had tested themselves either because they were symptomatic or as a part of the regular biweekly screening recommended by the organisation. All tests were performed using self-testing lateral flow devices for the detection of COVID-19 antigen. Specimens from HCWs were not sent to the reference laboratory. The vaccination status of the infected HCWs was not known, but it is likely that most were vaccinated as primary and booster vaccines were offered to all healthcare staff. None of the infected HCWs was admitted to the hospital.

In this report, we have described an outbreak of COVID-19 that occurred in a respiratory ward at a time when COVID-19 was very prevalent in the community with a rising number of hospitalisations. Many (8/14, 57%) of the patients in the ward were at high risk of severe COVID-19 despite vaccination [9]. A majority of the affected patients (12/14) also had underlying respiratory conditions (Table 1), but only 5/14 developed symptoms attributable to COVID-19 and only two of the infected patients had additional oxygen requirements. One patient was transferred to the intensive care unit for respiratory support but recovered with minimal intervention. Half of the patients (4/8) who were considered at high risk of severe COVID-19 received remdesivir and only one of the remaining six patients received remdesivir. Nearly all patients were discharged from the hospital, one died from COVID-19 pneumonitis, and on 31 July 2022, two patients were still in hospital for management of their underlying medical conditions, unrelated to COVID-19. Overall, the impact of COVID-19 on patients was much less severe than that described in earlier reports of HA-COVID-19 and hospital outbreaks with alpha and delta variants of SARS-CoV-2 where mortality rates of 27–40% were reported [7]. The reason for the reduced severity of COVID-19 in the outbreak described is not clear. The fact that most of the patients (12/14) were vaccinated against COVID-19 and had boosters as well may have played a role in reducing the severity while not preventing the infection with the Omicron variant [10]. It is also possible that the BA.5.2.1 variant, being more transmissible, may be less virulent than the alpha and delta variants. Finally, remdesivir and/or corticosteroids may have contributed to the reduction in the risk of adverse outcomes (e.g., need for mechanical ventilation/death). We could not establish the source of the infection. In the period before the outbreak, visitors were allowed on the ward but those with symptoms of COVID-19 were asked to exclude themselves. HCWs working or visiting the ward were expected to test themselves twice a week using tests for COVID-19 antigen and exclude themselves if positive. However, we could not confirm if all the visitors or HCWs were compliant with these recommendations. It is also possible that asymptomatic visitors or HCWs may have introduced the infection into the ward.

In conclusion, the low level of morbidity and mortality in a hospital-onset outbreak of COVID-19 caused by the BA.5.2.1 variant in a highly vaccinated vulnerable patient population suggests that either the vaccination is effective in reducing the severity of the illness or the BA.5.2.1 variant is less virulent or both. The role of remdesivir and/or corticosteroids in reducing the severity or mortality was not evaluated. It is conceivable that infection control measures in healthcare facilities could be revised when vaccinated patients are exposed to COVID-19. However, any changes in infection control measures should be carefully evaluated. It is likely that the findings of this outbreak are generalisable to other vaccinated hospitalised populations in the United Kingdom and Europe.

## Data Availability

Data pertaining to the outbreak is available from the authors.
